# Emergent *Escherichia coli* of the highly virulent B2-ST1193 clone producing KPC-2 carbapenemase in ready-to-eat vegetables

**DOI:** 10.1016/j.jgar.2024.11.020

**Published:** 2025-03

**Authors:** Karine Dantas, Gregory Melocco, Fernanda Esposito, Herrison Fontana, Brenda Cardoso, Nilton Lincopan

**Affiliations:** a Department of Microbiology, Institute of Biomedical Sciences, University of São Paulo, São Paulo, Brazil; b Department of Clinical Analysis, School of Pharmacy, University of São Paulo, São Paulo, Brazil; c One Health Brazilian Resistance Project (OneBR), São Paulo, Brazil

**Keywords:** WHO critical priority pathogens, Carbapenem resistance, Enterobacterales, Acid tolerance, Genomic surveillance

## Abstract

**Objectives:**

Critical priority carbapenem-resistant pathogens constitute a worldwide public health problem. *Escherichia coli* (*E. coli*) ST1193 is an emerging high-risk clone that demonstrates prolonged gut persistence, and association with community-onset urinary and bloodstream infections. The purpose of this study is to report microbiological and genomic data on the emergence of KPC-2-producing *E. coli* ST1193 in ready-to-eat (RTE) vegetables.

**Methods:**

RTE vegetables were purchased from markets in southeastern Brazil. Epiphytic and endophytic Gram-negative bacteria displaying resistance to broad-spectrum beta-lactams were identified by matrix-assisted laser desorption ionization-time of flight mass spectrometry. Whole-genome sequence was conducted using the Illumina NextSeq platform. Antimicrobial susceptibility, conjugation, and acid tolerance assays were performed. Virulence behaviour was evaluated using the *Galleria mellonella* (*G. mellonella*) infection model.

**Results:**

Epiphytic KPC-2-producing *E. coli* belonging to pandemic ST1193 was identified in RTE arugula. Genomic analysis predicted clinically relevant genes conferring resistance to β-lactams, fluoroquinolones, hazardous heavy metals, pesticides, disinfectants, and chlorine sanitizer. The *bla*_KPC-2_ gene was carried by a conjugative IncF plasmid. Acid tolerance of *E. coli* KPC-2/ST1193 during exposure to pH 2.0 was confirmed, being associated with *gadWX* and *ibaG* pH tolerance genes, supporting survival to stomach acid prior to reaching the small intestine, and potential for a dietary mode of host colonization. Virulent behaviour was supported by wide virulome of the highly virulent phylogroup B2, whereas single nucleotide polymorphisms of core genes (cgSNP)-based phylogenomics revealed clonal relationship with healthcare-associated lineages circulating in the United States, China, Mexico, France, and Brazil.

**Conclusions:**

We report the occurrence of KPC-2-producing *E. coli* of the highly virulent B2-ST1193 clone in RTE vegetables, highlighting a possible route of dissemination of the World Health Organization (WHO) priority pathogens to humans. © 2024 The Author(s). Published by Elsevier Ltd on behalf of International Society for Antimicrobial Chemotherapy.

## Highlights

• WHO critical priority pathogens in ready-to-eat vegetables have been investigated.
• Carbapenem-resistant *E. coli* was identified in RTE arugula sold in a supermarket.
• Genomic analysis revealed emergent *E. coli* ST1193 producing KPC-2 carbapenemase.
• *E. coli* KPC-2 displayed tolerance to acid pH and virulent behaviour in *G. mellonella*.
• Occurrence of *E. coli* KPC-2/ST1193 through ready-to-eat vegetables is discussed.

## 1 Introduction

The rapid spread of the World Health Organization (WHO) priority carbapenem-resistant pathogens is a public health problem no longer restricted to hospital settings [1]. In this regard, carbapenemase-producing *Escherichia coli* (*E. coli*) have begun to be detected in fresh vegetables [2], which is a critical issue, because many vegetables are now commercialized as ready-to-eat (RTE) foods [3]; being linked with outbreaks of foodborne diseases, and prone to transmit antimicrobial-resistant bacteria and/or their resistance genes to the human population [[3], [4]].

*E. coli* sequence type (ST) ST1193 is an emerging high-risk and fluoroquinolone-resistant clone that harbours mutations in DNA gyrase (GyrA-S83) and topoisomerase IV (ParC-S80), being associated with community-onset urinary and bloodstream infections worldwide [[5], [6], [7], [8]]. Studies confirm that the emergence of ST1193 represents the only known large clonal expansion of *E. coli* isolates apart from the global spread of ST131, over the last 2 decades, even replacing ST131 in certain regions [6]. Noteworthy, pandemic *E. coli* ST1193 demonstrate prolonged gut persistence with long-term colonization of healthy individuals, which is a critical issue because it is well-known that *E. coli* carried in the intestinal tract serves as a reservoir for endogen infections, mainly for urinary tract infections and bacteraemia [7,8].

Considering the high prevalence of KPC-2 in community-acquired infections in Latin American countries, studies on screening of carbapenemase-producing bacteria from fresh vegetables are urgently needed. In this study, as part of the Grand Challenges Explorations: New Approaches to Characterize the Global Burden of Antimicrobial Resistance Program, we have performed a local genomic surveillance of WHO critical-priority pathogens in RTE vegetables, alerting on the emergence of a highly virulent acid-tolerant KPC-2-producing *E. coli* of the international high-risk ST1193-B2 clone.

## 2 Materials and methods

Between 2021 and 2022, a total of 18 fresh RTE and bulk vegetable samples were purchased from markets in São Paulo, Brazil, the largest city in South America. As previously defined by the US Food and Drug Administration, the term “RTE” foods refers to “Any food that is normally eaten in its raw state or any other food, including a processed food, for which it is reasonably foreseeable that the food will be eaten without further processing that would significantly minimize biological hazards” [9]. Moreover, “RTE foods can be processed, packaged or unpackaged by traditional or industrial methods for immediate or later consumption” [10,11]. RTE foods include perishable vegetables and fruits sold in the marketplace for immediate consumption, without washing processes, as whole or fresh-cut products [11]. It was considered as “bulk,” fresh retail vegetables sold intact and not subjected to any treatment to eliminate pathogens. In fact, they are not indicated for consumption without washing processes, because a diverse range of human enteric pathogens can contaminate them [12]. Samples of lettuce (RTE, *n* = 4 and bulk, *n* = 4) and arugula (RTE, *n* = 6 and bulk, *n* = 4) were transported in the original packaging (RTE vegetables) or in sealed bags (bulk vegetables) in thermal boxes, being processed within 24 hours.

For the isolation of endophytic bacteria, samples were washed in running water and sanitized. For surface sanitization, ca. 5 g of leaves were immersed sequentially in 70% ethanol (for 1 min), sodium hypochlorite (2.5% chlorine, for 4 min), and 70% ethanol (for 30 s), and then washed 3 times in sterile distilled water [13]. Aliquots of the sterile water used in the final rinse were plated directly onto nutrient agar to confirm effectiveness of the sanitization protocol. Surface-sanitized leaves were macerated in 12 mL of sterile saline solution, then 1.0 mL of the macerate was transferred into tubes containing 4.0 mL of Luria Bertani sterile broth, and incubated at 37 °C with shaking at 90 rpm for 24 h. After this period, 10 µL of broth culture were plated on MacConkey agar supplemented with ceftriaxone (2 µg/mL) [13,14]. For the isolation of epiphytic bacteria, ca. 15 g of unwashed and unsanitized leaves were submerged in 150 mL of peptone 1% and shaken at 37 °C [15]. After 30 min, 1.0 mL was collected, inoculated into 4.0 mL of Luria-Bertani culture medium, and incubated at 37 °C with shaking at 90 rpm for 24 h. After this period, 10 µL was plated on MacConkey agar supplemented with ceftriaxone (2 µg/mL) [14]. After 24 h of incubation at 37 °C, colonies from epiphytic and endophytic isolation protocols were picked from the selective agar plates, subcultured, and streaked to obtain pure cultures with further species identification and antimicrobial susceptibility testing.

Bacterial identification was performed by time-of-flight matrix-assisted laser desorption mass spectrometry (MALDI-TOF/MS, Bruker). Antimicrobial susceptibility was performed by disc diffusion and Vitek 2 system (bioMérieux, France). The results were interpreted according to the Clinical and Laboratory Standards Institute [16].

For whole genome sequencing, genomic DNA was extracted using the PureLink™ Genomic DNA Mini Kit (Thermo Fisher Scientific, USA). DNA quality and concentration were assessed by NanoDrop spectrophotometer (Thermo Scientific) and Qubit® 2.0 fluorometer (Life Technologies, Carlsbad, CA), respectively. The library was constructed using the Nextera DNA Prep kit (Illumina, San Diego, CA) and sequenced using the NextSeq550 paired reads platform (2 × 75 bp). Raw sequencing data were quality filtered to remove low-quality bases (Phred quality < 20) using Trimmomatic version 0.32 (https://github.com/timflutre/trimmomatic). Quality-filtered reads were de novo assembled using CLC Genomics Workbench 12.0.3, and the sequences were annotated by PGAP version 3.2 (http://www.ncbi.nlm.nih.gov/genome/annotation_prok/).

Multilocus sequence typing (MLST), resistome, virulome, plasmids, and plasmid MLST (pMLST) were predicted using MLST 2.0 (https://cge.food.dtu.dk/services/MLST/), Resfinder 4.3 (http://genepi.food.dtu.dk/resfinder), VirulenceFinder 2.0 (https://cge.food.dtu.dk/services/VirulenceFinder/), PlasmidFinder 2.1 (https://cge.food.dtu.dk/services/PlasmidFinder/), and pMLST 2.0, respectively; from the Center for Genomic Epidemiology (https://genomicepidemiology.org/). Additionally, the phylogroup was determined using ClermonTyping (http://clermontyping.iame-research.center/). Genes of tolerance to disinfectants (quaternary ammonium compounds), pesticides (glyphosate), heavy metals, and chorine were predicted using ABricate (https://github.com/tseemann/abricate) through in silico comparative analysis with an internal database and the BacMet database (http://bacmet.biomedicine.gu.se/). Genes were predicted using a threshold of 95% for nucleotide identity and 100% for gene coverage. Genetic context analysis of the *bla*_KPC-2_ gene was performed against the clinical *Klebsiella pneumoniae* strain FCF3SP (ST442) (GenBank accession number: CP004367.2), using the Easyfig BioTool (https://bio.tools/easyfig).

For comparative phylogenomic analysis, publicly available genomes of 240 *E. coli* strains ST1193 were retrieved from National Center for Biotechnology Information (NCBI; https://www.ncbi.nlm.nih.gov/refseq/; Supplementary Table S1). For construction of the single nucleotide polymorphisms of core genes (cgSNP) phylogenetic tree, the genomes were annotated with Prokka software version 1.14.5 (https://github.com/tseemann/prokka) and the core genome was determined by Roary version 3.13.0 (https://github.com/sanger-pathogens/Roary). The cgSNP were identified and extracted by SNP-sites version 2.5.1 (https://github.com/sanger-pathogens/snp-sites). The phylogenomic tree was inferred by RAxML-NG version 1.1.0 (https://github.com/amkozlov/raxml-ng), using the general time reversible model of nucleotide substitution and gamma distribution with 100 bootstraps. Visualization and annotation of the topology of the tree were performed with iTol version 6 (https://itol.embl.de/).

For conjugation assays, donor and recipient strains (*E. coli* J53, sodium azide resistant) were co-cultured in a 1:1 ratio [13]. Transconjugants strains (J53_T_) were selected in MacConkey agar supplemented with 4.0 mg/mL of ertapenem and 100.0 mg/mL of sodium azide. The antibiotic resistance profile of J53_T_ strain was evaluated by disk-diffusion, and the polymerase chain reaction of the IncF plasmid and the *bla*_KPC-2_ gene was performed.

For acid tolerance assays, trypticase soy broth culture medium was prepared to cover acid pH scales ranging from 2.0 to 7.0. Volumes of 50 mL of trypticase soy broth were adjusted individually to a final pH of 2.0, 3.0, 4.0, 5.0, 6.0, and 7.0 by adding 0.1 M HCl, and using a pH meter [17]. Broths were sterilized and the pH was confirmed. Next, 2 mL of each broth at the different pH values were added in sterilized glass tubes. In brief, each tube was inoculated with bacterial cell suspension to a final concentration of 10^5^ colony forming unit (CFU), being incubated at 35 °C. After 1, 2, 6, and 24 h incubation, an aliquot (50 µL) of cell suspension was taken from each tube, diluted 1:10, 1:100, 1:1000, and 1:10,000, and cell viability was determined by plating 10 µL of each dilution on Trypticase soy agar plates and incubating for 24 h at 35 °C [13]. All assays were performed in duplicate.

For the evaluation of the virulence behaviour of the KPC-2-producing *E. coli* strain ST1193, the *Galleria mellonella* (*G. mellonella*) infection model was used [18]. The hypervirulent meningitis/sepsis-associated K1 *E. coli* MNEC RS218 and the *E. coli* ATCC25922 strain were used as virulent and non-virulent control, respectively [18]. Kaplan-Meier survival curve was plotted, and data were analysed by the log rank test, with *P* < 0.05 indicating statistical significance (Graph Pad Software, San Diego, CA).

## 3 Results

Twenty-eight epiphytic (*n* = 14) and endophytic (*n* = 14) Gram-negative bacteria were isolated from the screened vegetables (Supplementary Table S2). Whereas most isolates were identified as *Pseudomonas* spp., *Acinetobacter* spp., or *Stenotrophomonas maltophilia*, a carbapenem-resistant *E. coli* strain (REN5021) was isolated from a RTE packaged arugula sold in a supermarket. REN5021 displayed a multidrug-resistant profile [19], to aztreonam, ampicillin/sulbactam (minimum inhibitory concentration [MIC] ≥ 32 µg/mL), piperacillin/tazobactam (MIC ≥ 128 µg/mL), cefuroxime (MIC ≥ 64 µg/mL), ceftriaxone (MIC ≥ 64 µg/mL), cefotaxime, cefepime, ceftazidime, cefoxitin, ertapenem, imipenem (MIC ≥ 16 µg/mL), meropenem (MIC ≥ 16 µg/mL), nalidixic acid, ciprofloxacin (MIC ≥ 4 µg/mL), levofloxacin, enrofloxacin, trimethoprim-sulfamethoxazole and tetracycline, remaining susceptible to amikacin (MIC ≤ 1 µg/mL), gentamicin (MIC ≤ 1 µg/mL), and ceftazidime/avibactam.

Sequencing of the strain REN5021 resulted in a total of 4 224 911 reads, of which 6176 (0.15%) reads were removed after quality control (trimming). According to CheckM (https://github.com/Ecogenomics/CheckM), de novo assembly resulted in a genome completeness of 99.56% and 0.26% contamination. The genome size was calculated as 5 092 719 bp, assembled into 130 contigs, with 226.3 × coverage, and a G + C content of 50.64%, comprising with 4945 protein-coding sequences, 42 tRNAs, 4 rRNAs, and 202 pseudogenes.

Genomic analysis of carbapenem-resistant *E. coli* REN5021 confirmed the presence of genes conferring resistance to β-lactams (*bla*_KPC-2_ and *bla*_TEM-1B_), trimethoprim-sulfamethoxazole (*sul2* and *drfA*), tetracyclines (*tetB*), macrolides (*mphA*), and aminoglycosides [*aph(6)-Id, aph(3″)-Ib*]; as well as mutations in *gyrA* (S83L and D87N), *parC* (S80I) and *parE* (L416F) conferring resistance to fluoroquinolones. In addition, genes for heavy metals (*merR, merC*, and *tehB*), disinfectants (*emrD, tolC, mdtEF, tehA, acrEF*, and *emrK*), glyphosate herbicide (*phnPIEC*), and chlorine sanitizer (*yjiE, nemR*, and *rclRBC*) tolerance were also predicted. The *bla*_KPC-2_ gene was harboured by a Tn*4401b* transposon, within an IncF plasmid (pMLST K2:A1:B10), which was successfully transferred to the sodium azide-resistant *E. coli* J53 recipient strain. The overall comparison showed that this transposon exhibited high similarity with the Tn*4401b* transposon carried by a clinical *K. pneumoniae* ST442 (GenBank accession number: CP004367.2; Supplementary Fig. S1).

**Fig. 1:**
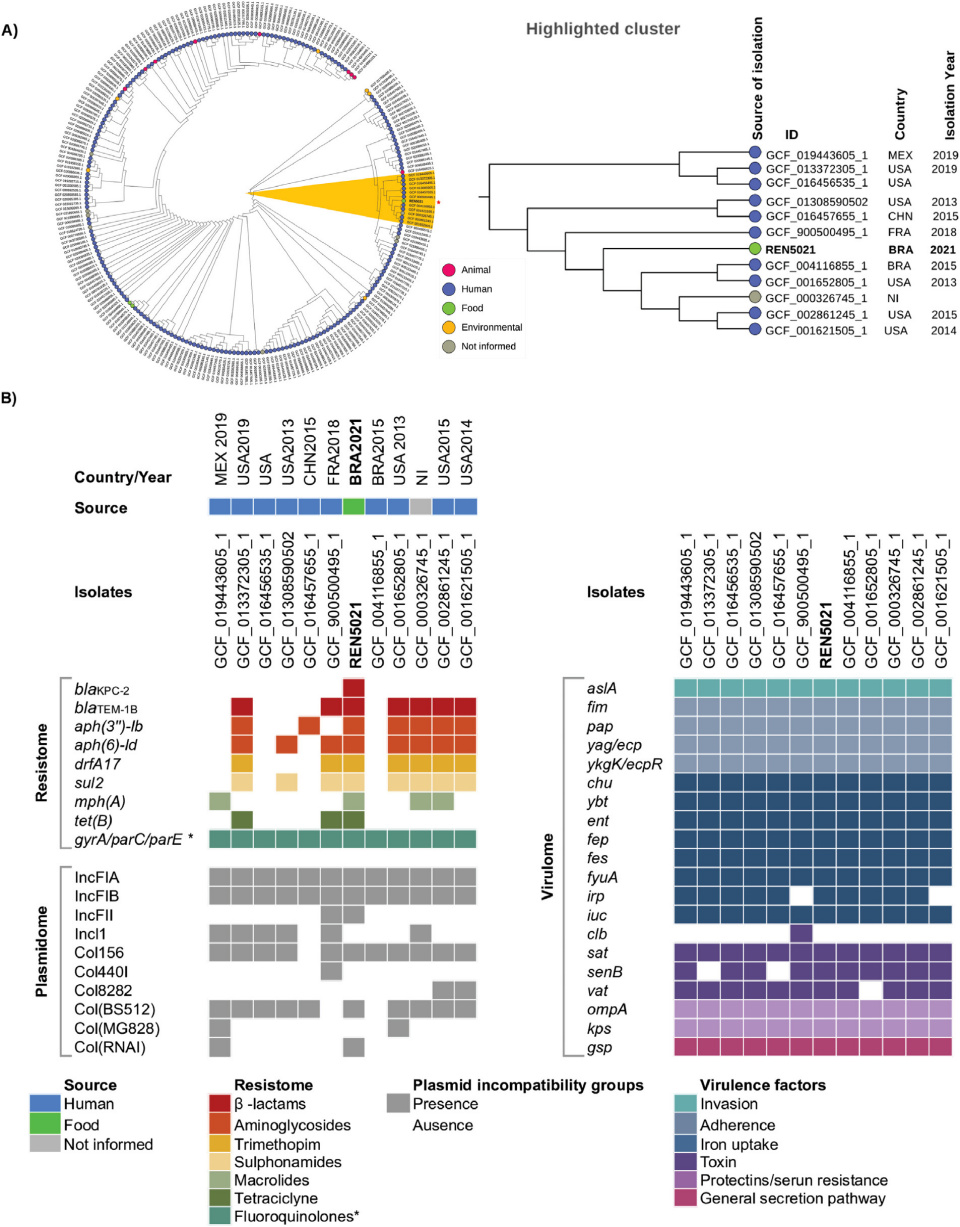
In A, core genome single nucleotide polymorphism (cgSNP)-based comparative phylogenomic analysis of epiphytic KPC-2-positive *Escherichia coli* (*E. coli*) strain REN5021, along with 240 publicly available genomes of international *E. coli* strains belonging to sequence type ST1193. The KPC-2-producing *E. coli* strain REN5021 was clustered (yellow highlight) along with healthcare-associated ST1193 *E. coli* lineages from the United States (51 – 969 SNP differences), Mexico (57 SNPs difference), France (93 SNPs difference), China (67 SNPs difference), and Brazil (62 SNPs difference). In B, heatmap showing comparative resistome, virulome and plasmidome of KPC-2-positive *E. coli* REN5021 and genomically related *E. coli* strains from the highlighted cluster in Figure 1A. The resistance genes are grouped by antimicrobial resistance classes, whereas virulence genes are listed according to their functions. Coloured squares represent the presence of genes. *, mutations; NI, not informed.

Whereas MLST analysis revealed that KPC-2-producing *E. coli* REN5021 belonged to the emergent high-risk fluoroquinolone-resistant clone ST1193 [[5], [6], [7], [8]], cgSNP-based phylogenomic analysis clustered *E. coli* REN5021 along with healthcare-associated ST1193 lineages from the United States (51 – 969 SNP differences), Mexico (57 SNPs difference), France (93 SNPs difference), China (67 SNPs difference), and Brazil (62 SNPs difference; Fig. 1A, Supplementary Table S3). Moreover, resistome, virulome and plasmidome of these genomically related strains confirm that ST1193 is a global fluoroquinolone-resistant ExPEC+/UPEC+ clone that has now acquired the *bla*_KPC-2_ gene, spreading beyond hospital walls in Brazil (Fig. 1B).

Noteworthy, genes related to survival at acid pH (*ibaG* and *gadWX)* were identified. In this regard, tolerance of *E. coli* REN5021 to pH values ranging from 2.0 to 7.0 was confirmed with persistence of bacterial cells for up to 6 h of incubation at pH 3.0 and 4.0, whereas, after 24 h at pH 3.0, no viable cells were observed. At pH 2.0, there was a reduction of 3 log CFU/mL after 2 h of incubation, supporting that REN5021 must survive stomach acid prior to reaching the small intestine, where pH ranges from 6.5 to 7.5 [20]. However, after 6 h of incubation at pH 2.0, no viable cells were observed.

**Fig. 2:**
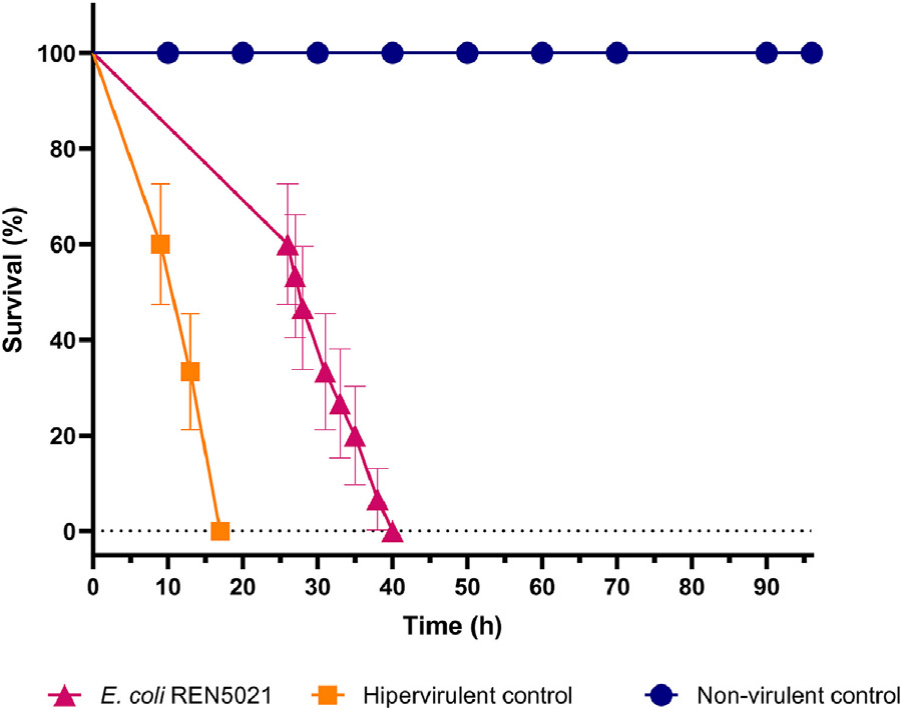
Kaplan-Meier survival curves of *Galleria mellonella* larvae. Survival was monitored after infection with 10^5^ colony forming unit (CFU)/larva of: (i) KPC-2-producing *Escherichia coli* (*E. coli*) ST1193 (strain REN5021), isolated from ready-to-eat (RTE) vegetables; (ii) *E. coli* ATCC 25 922 (non-virulent control) and; (iii) *E. coli* MNEC (hypervirulent control). Data were analysed by the log rank test, with *P* < 0.05 indicating statistical significance (Graph Pad Software, San Diego, CA).

*E. coli* REN5021 displayed a wide virulome correlated with the highly virulent phylogroup B2, with presence of genes encoding siderophores (yersiniabactin, *ybt* cluster; and enterobactin, *ent* cluster), toxins (*sat, senB*, and *vat*), and K1 capsule (*kpsDTM*) production. In this regard, a highly virulent behaviour (*P* < 0.05) of KPC-2-positive *E. coli* was observed in the *G. mellonella* model, leading to 100% mortality of the larvae within 40 h of infection, when compared (*P* < 0.05) to the non-virulent control, which did not kill any of the larvae during the 96 h of observation (Fig. 2). In contrast, infection with the hypervirulent control (*E. coli* MNEC) resulted in 100% mortality of the larvae within 24 h.

## 4 Discussion

In our study, RTE and bulk vegetable samples were screened for the presence of broad-spectrum cephalosporin- and carbapenem-resistant Gram-negative bacteria. Although there was a predominance of *Pseudomonas* spp. and *Acinetobacter* spp., the occurrence of the global ST1193/B2 clone of carbapenem-resistant *E. coli*, carrying a conjugative IncF/*bla*_KPC-2_ plasmid, in an RTE vegetable was highlighted, because this profile is classified as critical priority by the WHO [1]. In this regard, *Pseudomonas* and *Acinetobacter* species are commonly present in the microbiota of fresh vegetables [21], whereas the occurrence of *E. coli* in food is related to faecal contamination, because this bacterium is the most abundant facultative anaerobe of intestinal tracts of humans and other mammals [22]. Therefore, presence of *E. coli* in RTE foods is undesirable because it suggests poor hygienic conditions that lead to contamination [3].

Although some studies have recently reported critical priority pathogens in fresh vegetables sold in Latin America [[13], [15]], its occurrence in RTE products has not been reported, so far, alerting on a critical public health problem. Indeed, consumption of fresh RTE vegetables has increased globally, contributing to outbreaks and cases of foodborne illness [3,4]. In this respect, presence of acid pH tolerance genes *ibaG* and *gadWX* and survival of KPC-2-positive *E. coli* REN5021 at pH values as low as the stomach support a potential for a dietary mode of host colonization [23,24].

Acid resistance in *E. coli* is defined as the ability to withstand an acid challenge of pH 2.5 or less [25]. Acid resistance is an important property, because it enables *E. coli* to survive gastric acidity and volatile fatty acids produced as a result of intestinal fermentation. In fact, resistance to acid stresses is necessary for colonization and for establishing a commensal relationship with mammalian hosts [25].

Another clinical and epidemiologically relevant result is that *E. coli* REN5021 belonged to the ST1193, an international high-risk clone related to human and animal infections [[5], [6], [7], [8]]. In Brazil, although *E. coli* ST1193 has been circulating since at less 2015, its isolation has been associated with intestinal colonization and urinary tract infections of inpatients (Genome Assembly number: GCF_004116855.1), whereas no carbapenem-resistant strain has been documented so far.

Despite the limited number of samples analysed, with a single critical-priority pathogen being isolated and genomically characterized in this study, the occurrence of any clinically relevant pathogen resistant to carbapenems and clonally related to hospital lineages, in RTE vegetables, should be considered abnormal and undesirable, becoming a public health risk that deserves continued surveillance. In this regard, the number of reports of carbapenem-resistant bacteria in fresh vegetables and agricultural soil has increased in recent years [[26], [27], [28], [29]], highlighting the need for better management in the production systems of RTE vegetables, as they will not undergo additional sanitization processes after purchase. On the other hand, surveillance programs to establish the extent of contamination of commercial vegetables could help in understanding the real contribution of these foods in the dissemination of the WHO critical-priority pathogens and their resistance genes.

## Appendix Supplementary materials

Image, application 1

Image, application 2

Image, application 3

Image, application 4
