# Supplementary material for: Emergent Escherichia coli of the highly virulent B2-ST1193 clone producing KPC-2 carbapenemase in ready-to-eat vegetables
Source: J Glob Antimicrob Resist. 2025 Mar;41:105–10. doi: 10.1016/j.jgar.2024.11.020 (PMC11888992; doi:10.1016/j.jgar.2024.11.020)
Supplement: Supplementary file 2 [file mmc2.doc]

| **Supplementary Table S2.** Antibiotic resistance profile of epiphytic and endophytic bacteria isolated from bulk or ready-to-eat vegetables | | | |
| --- | --- | --- | --- |
| Sample^a^ | Specie/Strain | Location^b^ | Antibiotic resistance profile^c^ |
| Fresh vegetables | | | |
| ALF1 | *Pseudomonas guariconensis* AGN201^d^ | 23°33'34.1"S 46°38'34.6"W | ATM, ENO |
| ALF1 | *Pseudomonas putida* AGN301^e^ | 23°33'34.1"S 46°38'34.6"W | ATM |
| ALF2 | *Pseudomonas putida* AGN2021 ^d^ | 23°33'56.9"S 46°41'32.6"W | ATM |
| RUC1 | *Pseudomonas putida group* RGN301^e^ | 23°33'56.1"S 46°38'50.3"W | ATM, CAZ, LVX |
| RUC2 | *Acinetobacter vivianii* RGO2012^d^ | 23°33'21.9"S 46°41'12.5"W | CRO, CTX, CAZ, CIP |
| RUC2 | *Acinetobacter calcoaceticus* RGO3011^e^ | 23°33'21.9"S 46°41'12.5"W | CRO, CAZ, CTX |
| RUC2 | *Pseudomonas putida* RGO2011^d^ | 23°33'21.9"S 46°41'12.5"W | ATM, CAZ, MER, CIP, LVX, ENO |
| RUC3 | *Pseudomonas monteilii* RGO3011^d^ | 23°33'51.9"S 46°41'03.7"W | ATM, CAZ, CPM, CIP, LVX |
| RUC3 | *Acinetobacter calcoaceticus* RGO2023^d^ | 23°33'51.9"S 46°41'03.7"W | CRO, CTX, CAZ, CIP, SUT |
| RUC3 | *Acinetobacter calcoaceticus* RGO3022^e^ | 23°33'51.9"S 46°41'03.7"W | CRO, CTX, CAZ |
| RUC4 | *Pseudomonas aeruginosa* RGH201^d^ | 23°33'11.6"S 46°39'09.2"W | - |
| RUC4 | *Pseudomonas putida* RGH301^e^ | 23°33'11.6"S 46°39'09.2"W | - |
| Ready-to-eat vegetables | | | |
| ALF6 | *Pseudomonas chlororaphis* AEO2011^d^ | 23°33'44.5"S 46°39'03.4"W | - |
| ALF7 | *Acinetobacter calcoaceticus* AEO4031^d^ | 23°33'51.9"S 46°41'03.7"W | CRO, CTX, CAZ, CPM, CIP |
| ALF8 | *Pseudomonas aeruginosa* AEH2011^d^ | 23°33'49.0"S 46°41'24.8"W | ATM, CAZ, CPM |
| RUC5 | *Pseudomonas mosselii* REN201^d^ | 23°33'11.6"S 46°39'09.2"W | ATM, CPM, LVX, CIP, ENO |
| RUC5 | *Pseudomonas aeruginosa* REN301^e^ | 23°33'11.6"S 46°39'09.2"W | ATM, CAZ, ENO |
| RUC6 | *Pseudomonas mosselii* REN2024 ^d^ | 23°33'51.9"S 46°41'03.7"W | ATM, CAZ, CPM, CIP; LVX, ENO |
| RUC6 | *Pseudomonas putida* REN301^e^ | 23°33'51.9"S 46°41'03.7"W | ATM, LVX, ENO |
| RUC6 | *Pseudomonas aeruginosa* REN3022^e^ | 23°33'51.9"S 46°41'03.7"W | ATM, ENO |
| RUC6 | *Escherichia coli* REN5021^d^ | 23°33'51.9"S 46°41'03.7"W | ATM, SAM, TZP, CRX, CRO, CTX, CAZ, CPM, CFO, ETP, IPM, MER, NAL, CIP, LVX, ENO, SUT, TET |
| RUC7 | *Acinetobacter baumannii* REN2031^d^ | 23°33'51.9"S 46°41'03.7"W | CRO, CTX |
| RUC7 | *Acinetobacter soli* REN2032^d^ | 23°33'51.9"S 46°41'03.7"W | CRO, CTX |
| RUC7 | *Acinetobacter pittii* REN303^e^ | 23°33'51.9"S 46°41'03.7"W | CRO, CTX |
| RUC7 | *Pseudomonas fulva* REN3032A^e^ | 23°33'51.9"S 46°41'03.7"W | ATM |
| RUC8 | *Stenotrophomonas maltophilia* REO2011^d^ | 23°33'51.9"S 46°41'03.7"W | - |
| RUC8 | *Stenotrophomonas maltophilia* REO3011^e^ | 23°33'51.9"S 46°41'03.7"W | - |
| RUC8 | *Acinetobacter radioresistens* REO2015^d^ | 23°33'51.9"S 46°41'03.7"W | CRO |
| RUC8 | *Acinetobacter baylyi* REO3012^e^ | 23°33'51.9"S 46°41'03.7"W | CRO, CTX |
| ^a^ ALF, lettuce; RUC, arugula.  ^b^ Sample location  ^c^ATM, aztreonam; SAM, ampicillin/sulbactam; TZP, piperacillin/tazobactam, CRX, cefuroxime; CRO, ceftriaxone; CTX, cefotaxime; CAZ, ceftazidime; CPM, cefepime; CFO, cefoxitin; ETP, ertapenem; IPM, imipenem; MER, meropenem; NAL, nalidixic acid; CIP, ciprofloxacin; LVX, levofloxacin; ENO, enrofloxacin; SUT, trimethoprim/sulfamethoxazole; TET, tetracycline.  ^d^ Epiphytic.  ^e^ Endophytic. | | | |
