# Supplementary material for: Emergent Escherichia coli of the highly virulent B2-ST1193 clone producing KPC-2 carbapenemase in ready-to-eat vegetables
Source: J Glob Antimicrob Resist. 2025 Mar;41:105–10. doi: 10.1016/j.jgar.2024.11.020 (PMC11888992; doi:10.1016/j.jgar.2024.11.020)
Supplement: Supplementary file 3 [file mmc3.doc]

**Supplementary material**

**FIG S1**

**
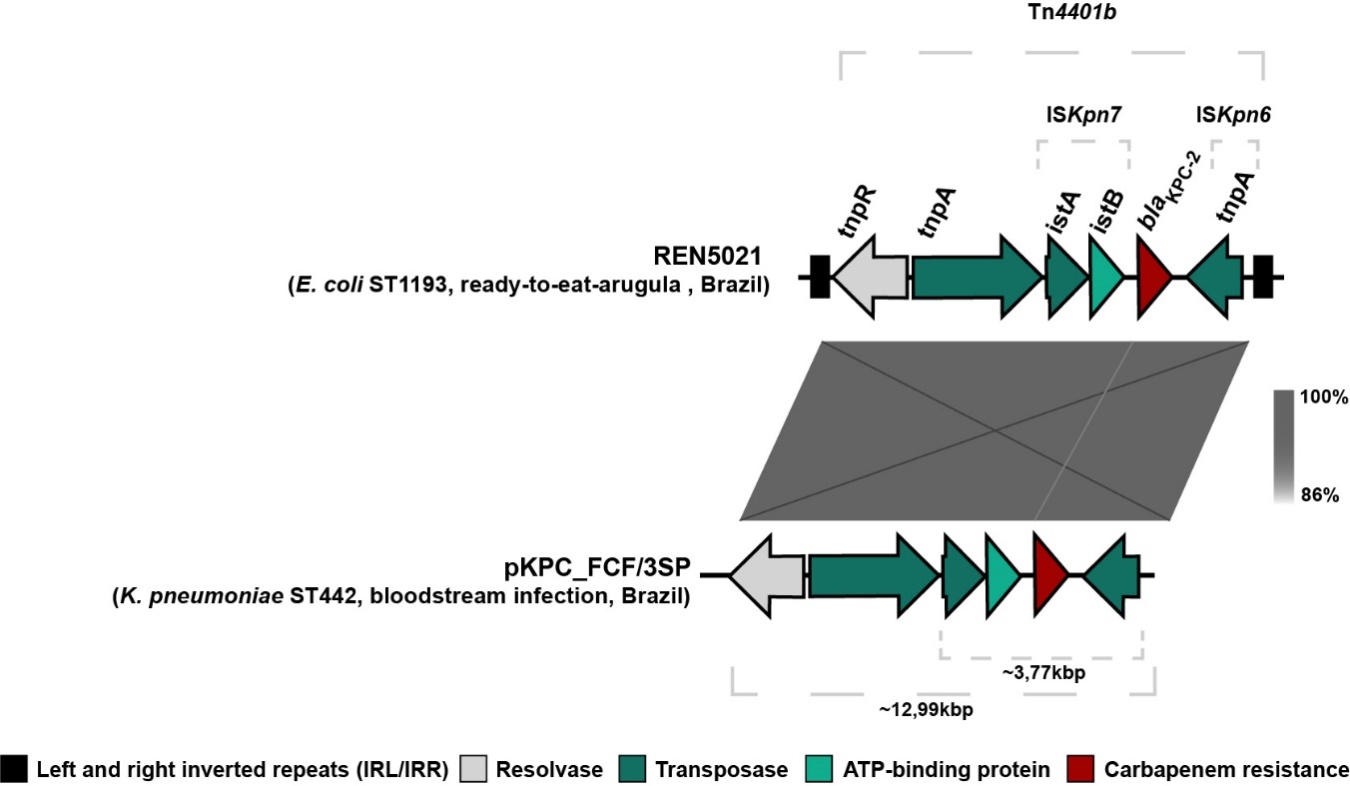
**

**FIG S1** Overall comparison of Tn*4401b* containing the *bla*_KPC-2_ gene carried by *E. coli* REN5021 strain belonging to ST1193, isolated from ready-to-eat vegetable, and clinical *K. pneumoniae* FCF3SP strain belonging to ST442. The Tn*4401b* was composed of genes encoding to resolvase (*tnpR,* grey triangles), transposase (*tnpA* and *istA*, dark-green triangles), ATP-binding protein (*istB*, light-green triangles) and carbapenem resistance (*bla*_KPC-2_, red triangles). The *bla*_KPC-2_ was flanked by a ~3.77 kb region composed of IS*Kpn7*-*bla*_KPC-2_ -IS*Kpn6*. Black rectangles represent left and right inverted repeats (IRL and IRR, respectively) delimiting Tn*4401b*. Comparative environments of *bla*_KPC-2_ gene were performed using Easyfig (<https://bio.tools/easyfig>).
